# Examining the neurodevelopmental and motor phenotypes of Bohring-Opitz syndrome (ASXL1) and Bainbridge-Ropers syndrome (ASXL3)

**DOI:** 10.3389/fnins.2023.1244176

**Published:** 2023-11-06

**Authors:** Maya C. Ayoub, Jeffrey T. Anderson, Bianca E. Russell, Rujuta B. Wilson

**Affiliations:** ^1^Division of Child Neurology, Department of Pediatrics, UCLA Health, Los Angeles, CA, United States; ^2^Department of Medicine, UCLA Health, UCLA David Geffen School of Medicine, Los Angeles, CA, United States; ^3^Division of Clinical Genetics, Department of Human Genetics, UCLA Health, UCLA David Geffen School of Medicine, Los Angeles, CA, United States; ^4^Division of Child Psychiatry, Department of Psychiatry, Semel Institute for Neuroscience and Human Behavior, UCLA David Geffen School of Medicine, Los Angeles, CA, United States

**Keywords:** Bohring-Opitz syndrome, ASXL1, Bainbridge-Ropers syndrome, ASXL3, chromatin modifying disorders, autism

## Abstract

**Background:**

Chromatin Modifying Disorders (CMD) have emerged as one of the most rapidly expanding genetic disorders associated with autism spectrum disorders (ASD). Motor impairments are also prevalent in CMD and may play a role in the neurodevelopmental phenotype. Evidence indicates that neurodevelopmental outcomes in CMD may be treatable postnatally; thus deep phenotyping of these conditions can improve clinical screening while improving the development of treatment targets for pharmacology and for clinical trials. Here, we present developmental phenotyping data on individuals with Bohring-Optiz Syndrome (BOS – ASXL1) and Bainbridge-Ropers Syndrome (BRS – ASXL3) related disorders, two CMDs highly penetrant for motor and developmental delays.

**Objectives:**

To phenotype the motor and neurodevelopmental profile of individuals with ASXL1 and ASXL3 related disorders (BOS and BRS). To provide a preliminary report on the association of motor impairments and ASD.

**Methods:**

Neurodevelopmental and motor phenotyping was conducted on eight individuals with pathogenic ASXL1 variants and seven individuals with pathogenic ASXL3 variants, including medical and developmental background intake, movement and development questionnaires, neurological examination, and quantitative gait analysis.

**Results:**

Average age of first developmental concerns was 4 months for individuals with BOS and 9 months in BRS. 100% of individuals who underwent the development questionnaire met a diagnosis of developmental coordination disorder. 71% of children with BOS and 0% of children with BRS noted movement difficulty greatly affected classroom learning. Participants with BRS and presumed diagnoses of ASD were reported to have more severe motor impairments in recreational activities compared to those without ASD. This was not the case for the individuals with BOS.

**Conclusion:**

Motor impairments are prevalent and pervasive across the ASXL disorders with and without ASD, and these impairments negatively impact engagement in school-based activities. Unique neurodevelopmental and motor findings in our data include a mixed presentation of hypo and hypertonia in individuals with BOS across a lifespan. Individuals with BRS exhibited hypotonia and greater variability in motor skills. This deep phenotyping can aid in appropriate clinical diagnosis, referral to interventions, and serve as meaningful treatment targets in clinical trials.

## 1. Introduction

Chromatin Modifying Disorders (CMD), also titled Mendelian disorder of the epigenetic machinery, are caused by variants affecting proteins responsible for chromatin regulation through joint or singular functions of reading, writing, modifying, and erasing (Fahrner and Bjornsson, 2019). Their association with autism spectrum disorders (ASD) is emerging through large scale genomics research (Lasalle, 2013; De Rubeis et al., 2014; Fahrner and Bjornsson, 2019). Two specific CMDs discussed here include Bohring-Opitz Syndrome (BOS), caused by truncating variants in ASXL1, and Bainbridge-Ropers Syndrome (BRS), caused by truncating variants in ASXL3. Though not covered in this publication, truncating variants in ASXL2 are associated with Shashi-Pena Syndrome. All three are neurodevelopmental conditions with variable associated congenital anomalies.

The three ASXL genes (additional sex combs-like 1, 2, and 3), play a critical role in embryonic development and reading of posttranslational histone modifications (Fisher et al., 2003; Sanchez and Zhou, 2011; Aravind and Iyer, 2012; Katoh, 2015; Jain et al., 2020). They are known to direct histone modification through polycomb repressive complex 1 and 2 (PRC1/−2) and the polycomb repressive deubiquitinase (PR-DUB) complex (Pereira et al., 2010). ASXL1 mutations have recently been linked to activation of the Wnt signaling pathway (Lin et al., 2023). The *Drosophila melanogaster* ortholog, *Asx*, is known to interact with Hox genes and is responsible for body patterning in flies (Sinclair et al., 1992). The exact mechanism of action of disease in the ASXL related disorders remains under investigation (Lin et al., 2023).

BOS has been described clinically since 1975 (Oberklaid and Danks, 1975) and was molecularly defined in 2011 when truncating ASXL1 variants were first associated with BOS (Holschen et al., 2011). There is a common set of facial features associated with BOS including widely-set eyes with prominent globes, full cheeks with facial hypotonia, palate anomalies, and micrognathia. A distinctive “BOS” posture (elbow, wrist, and metacarpopharyngeal joint flexion with ulnar deviation) with mixed hypotonia and hypertonia is well described in infancy/early childhood (Bohring et al., 1999; Russell et al., 2018). Degrees of intellectual disability are variable, but most individuals have severe to profound impairments, and seizures are common. Based on a review of reported case series and case reports, some individuals with BOS have been able to establish assisted gait with walkers or braces, though most are unable to walk independently (Russell et al., 2018). A specific genotype to phenotype correlation has not been identified (Russell et al., 2018, 2023). The largest cohort of individuals with BOS includes 39 participants, with clinical data provided via survey from caregivers. Motor data from this cohort indicates significant delays in developmental milestones such as rolling, sitting and walking (Russell et al., 2023).

Unlike BOS, BRS was not described until 2013 when Bainbridge et al. reported ASXL3 truncating variants in connection to a novel neurodevelopmental condition (Bainbridge et al., 2013). Clinical presentation includes nonspecific facial features, typically moderate to severe intellectual disability or developmental delays, autistic features, and speech/language delays. Individuals with BRS have been described to have early hypotonia with transition to spasticity – the posture is distinctive from BOS posture, with flexion at the elbows, wrists, and fingers (Balasubramanian and Schirwani, 2020). The most comprehensive phenotyping study (Schirwani et al., 2021), reported 83% with known hypotonia, and only 11% of individuals with gait at or before 18 months of age. A subsequent case series proposed a potentially milder physical, intellectual, and motor phenotype with familial inheritance (Schirwani et al., 2023).

Phenotypically, the motor impairments which are prevalent in BOS and BRS may play a role in the neurodevelopmental phenotype. Motor development in early childhood is fundamental in shaping how the child experiences their environment. These experiences lay the foundation for advances in perception, cognition, language, and social interaction (Piaget, 1952; Thelen, 2000; Iverson, 2010). Delayed or impaired motor development can have a negative cascading effect on multiple developmental domains. Motor impairments are highly prevalent and pervasive in individuals with ASD with a known genetic syndrome, and have been related to severity of genetic mutations (Fournier et al., 2010; Bishop et al., 2017; Buja et al., 2018; Wilson et al., 2020). A detailed study of Duplication 15q, a genetic syndrome highly penetrant for motor impairments and ASD, found that although all individuals with this syndrome met criteria for ASD on the Autism Diagnostic Observation Schedule (ADOS-2), they showed relative strength in social interest and behaviors that did not require sustained motor control. Thus, it was hypothesized that motor impairments likely influence the social communication abilities of these individuals, and more detailed evaluation of each of these domains would be important to better understand this relationship (DiStefano et al., 2016).

Given the degree of motor impairments and neurodevelopmental diagnoses that have been described through caregiver report and case series in BOS and BRS, deep phenotyping of the motor domain and examining the association with ASD may yield meaningful information about these conditions. There has been recent evidence that atypical neurologic pathways may be treatable postnatally in CMDs (Alarcón et al., 2004; Korzus et al., 2004; Guy et al., 2007; Fahrner and Bjornsson, 2019). Utilizing quantitative and qualitative techniques to improve the understanding of the clinical presentation of these conditions can inform natural history studies and potential treatment targets in clinical trials. In this study, we conducted motor and neurological phenotyping of two CMDs, BOS and BRS. We then conducted a preliminary analysis of how motor impairments in these conditions may impact other areas of functioning and how they may be associated with a presumed ASD diagnosis.

## 2. Methods

### 2.1. Participants

Eight individuals with BOS and nine with BRS underwent extensive phenotyping at a family conference, consisting of the following questionnaires (completed by caregivers) and testing (completed by two of the authors of the study). Two individuals with a reported mutation of ASXL3 were removed due to lack of verification of genetic testing reports.

### 2.2. Procedures

All research was approved by the UCLA Institutional Review Board (IRB#18–000280). Informed consent was obtained from caregivers prior to data collection. Recruitment and data collected was conducted at the UCLA David Geffen School of Medicine and the ARRE Foundation ASXL Scientific and Family Conference (Located in the Luskin Conference Center, Los Angeles California, from July 22–23, 2022).

### 2.3. Measures

#### 2.3.1. Clinical developmental history and medical questionnaire

Caregivers completed a comprehensive medical background form with questions focusing on pregnancy and neonatal history; developmental, behavioral, and social milestones; concurrent diagnoses (cerebral palsy, epilepsy, neurodevelopmental disorders); presence of a diagnosis of ASD and details of diagnosis (provider type/medical center where diagnosis was made); and prior neurodiagnostic work-up (genetic testing, imaging, etc).

#### 2.3.2. Movement assessment

Caregivers completed the Movement Assessment Battery for Children (MABC) checklist – a standardized measure of motor function examining manual dexterity, aiming, catching, and balance (Banátová and Psotta, 2022).

#### 2.3.3. Developmental coordination

Caregivers completed the Developmental Coordination Disorder Questionnaire (DCDQ) - a measure of functional activities (Wilson et al., 2009).

#### 2.3.4. Neurologic examination

Participants underwent a standardized neurological examination by a behavioral child neurologist (author on this study-RBW). The study examination included characteristic physical features, cranial nerve exam, musculoskeletal exam, neurocutaneous exam, examination of tone, examination of muscle strength, cerebellar exam (including coordination, ataxia, and gait), sensory exam, and reflexes.

#### 2.3.5. Gait assessment

Participants underwent quantitative gait analysis via the Zeno electronic walkway (Protokinetics, Havertown, PA) in conjunction with the ProtoKinetics Movement Analysis Software (PKMAS). Two participants did not complete the quantitative gait analysis due to being non-ambulatory. Three participants that attempted a quantitative gait analysis trial were ultimately excluded as they required extensive support for ambulation, affecting the interpretation of the spatiotemporal gait variables. Participants walked four full lengths of the 16 ft. mat, starting and ending each pass with several feet of space on either side of the mat to account for acceleration and deceleration. Each pass consisted of spontaneous self-pace walking in which participants produced and controlled their own natural speed. Additional passes were collected in cases where a participant stepped off the mat or their natural speed was not maintained. The UCLA research team observed all trials and removed any footfalls that did not fall entirely on the mat or did not represent the participant’s natural gait. Only passes that contained five or more consecutive valid footfalls were included in the final analysis. The spatiotemporal gait variables are presented in three domains as follows: 1. Pace: cadence (steps/min), step length (centimeters [cm]), normalized step length, velocity (cm/s), normalized velocity (1/s). 2. Postural control: stride width (cm), normalized stride width. 3. Variability: step length coefficient of variation (%), step time coefficient of variation (%), and stride width standard deviation (cm). The eight self-paced gait trials were averaged together to provide a global mean of all gait trials (Wilson et al., 2020).

#### 2.3.6. Data storage and analysis

Results were stored securely in RedCap, with data visualization via Excel and Prism Software.

## 3. Results

Demographic information is presented in Table 1.

**Table 1:** Subject demographics.

BOS (ASXL1)	Subject 1	Subject 2	Subject 3	Subject 4	Subject 5	Subject 6	Subject 7	Subject 8
Sex	*M*	*M*	*F*	*F*	*F*	*F*	*M*	*M*
Age (years)	11	6	5	11	9	18	6	8
Race	White	White	Black or African American	White	White	White	Asian	White
Hispanic/latinx descent	*N*	*N*	*Y*	*N*	*N*	*Y*	*N*	*N*
Genetic diagnosis	ASXL1: c.2922C > A p.Y974X	ASXL1: c.4243C > T p.R1415X	ASXL1: c.3202C > T p.R1068X	ASXL1: c.3077del p.G1026Dfs*21	ASXL1: c.2981delC p.P994Lfs*30	ASXL1: c.2013_2014del p.C672WfsX4	ASXL1: c.4196dupT p.L1399FfsX25 + GJB2 related hearing loss	ASXL1: c.1867C > T p.Q623X

BRS (ASLX3)	Subject 9	Subject 10	Subject 11	Subject 12	Subject 13	Subject 14	Subject 15
Sex	*M*	*M*	*F*	*F*	*F*	*F*	*F*
Age	5	4	4	9	3	3	5
Race	White	Asian	White	White	White	White	White
Hispanic/latinx descent	*N*	*Y*	*N*	*N*	*N*	*N*	*N*
Genetic Diagnosis	ASXL3: c.3964C > T p.Q1322X	ASXL3: c.4120_4123dupATAG p.A1375Dfs*7	ASXL3: c.4788_4816delinsT p.C1597GfsTer9	ASXL3: c.4648A > T p.K1550X	ASXL3: c.4060_4061delTC: p.S1354HfsX2	ASXL3: c.4034_4035dup p.I1346Pfs*15	ASXL3: c.1698_1699delGA p.E566DfsX20

Demographic data divided by subject group.

### 3.1. Clinical developmental history and medical questionnaire

This questionnaire was completed by 8/8 BOS participants and by 7/7 BRS participants. In the developmental history it was found that the average age of first developmental concerns was 4 months in BOS and 9 months in BRS. In BOS, 6/8 participants had a stay in the Neonatal Intensive Care Unit (NICU) for various medical reasons. In BRS, 0/7 participants had a stay in the NICU. 4/8 BOS participants achieved locomotion, with average age of first steps at 40 months; in BRS, average age of first steps was 27 months (one participant removed for “unknown” first age of walking 10 steps). 25% of BOS participants and 29% of BRS participants had a presumed diagnosis of ASD. Diagnoses were reported to be performed at academic medical centers (Doernbecher Autism Clinic at Doernbecher Children’s Hospital, University of Michigan, Children’s Hospital of Philadelphia, and Seattle Children’s). The provider types were either developmental behavioral pediatrician or psychologist. See Table 2 for clinical characteristics.

**Table 2:** Clinical developmental history.

	# of subjects with ASXL1 out of sample (%)	# of subjects with ASXL3 out of sample (%)
Age of first developmental concerns		
0 months	3/8 (38%)	0/7 (0%)
2–6 months	3/8 (38%)	2/7 (29%)
8–12 months	2/8 (25%)	5/7 (71%)
Parent report of ASD diagnosis		
	2/8 (25%)	2/7 (29%)
Prevalence of epilepsy diagnosis		
	5/8 (63%)	0/7 (0%)
Prevalence of cerebral palsy diagnosis		
	2/7 (29%)	2/7 (29%)

Clinical data for subjects with ASXL1 (BOS) and ASXL3 (BRS).

### 3.2. MABC

All BOS participants with a fully graded MABC (*n* = 6) had severe motor impairments in all motor domains (scores falling within the red zone once graded for age). All BRS participants also had severe motor impairments in all motor domains (*n* = 3 participants with fully graded MABC checklists whose age fell within the appropriate grading range). 71% of BOS and 0% of BRS noted movement difficulty “greatly” affected classroom learning. Participants with BRS and ASD showed more severe motor impairments in recreational activities compared to BRS and No-ASD (see Table 3). This was not the case in BOS.

**Table 3:** MABC.

	Sample size	Classroom learning	PE/recreational activities	Self esteem	Social interaction
BOS (ASXL1)	7	71% (20% of these individuals have ASD)	100% (29% of these individuals have ASD)	29% (50% of these individuals have ASD)	71% (40% of these individuals have ASD)
BRS (ASXL3)	5	0%	40% (100% of these individuals have ASD)	0%	0%

Parents responding that motor difficulties adversely affect the child’s categories of learning/social interaction “a great deal” on the movement assessment battery for children checklist.

### 3.3. DCDQ

**Figure 1:**
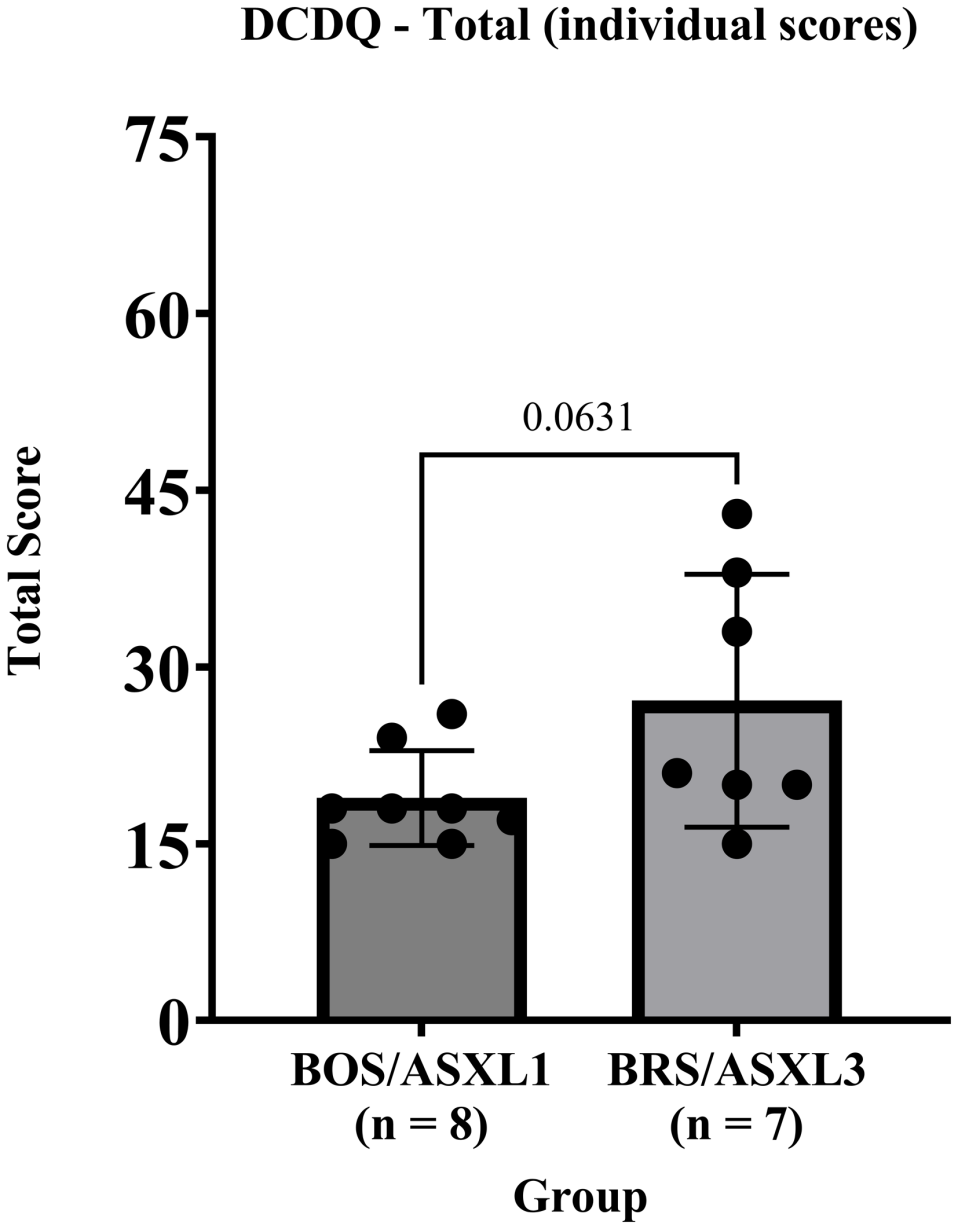
DCDQ total scores for BOS (ASXL1) and BRS (ASXL3).

We found that on the DCDQ, all participants with BOS and BRS met an indication of developmental coordination disorder, although 5 individuals were outside of the validated age range for this measure (refer to Figure 1 for individual score totals). BOS participants exhibited a tighter or equivalent range of scores on all portions of the DCDQ assessment compared to participants with BRS (see Table 4).

**Table 4:** DCDQ.

Portion of DCDQ	BOS (ASXL1) *n* = 8 participants	BRS (ASXL3) *n* = 7 participants
Control during movement – out of 30 points	6 to 11	6 to 18
Fine motor/handwriting – out of 20 points	4	4 to 12
General coordination – out of 25 points	5 to 13	5 to 13

Range of scores on the DCDQ. Lower scores indicating the presence or suspicion of a developmental coordination disorder.

### 3.4. Neurologic examination

**Figure 2:**
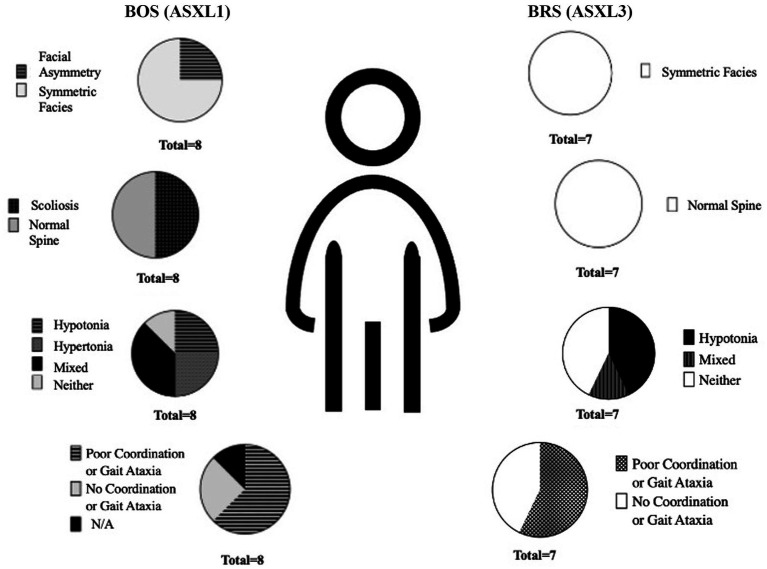
Percent of physical exam findings noted on participants with BOS (ASXL1) and BRS (ASXL3).

3 out of 8 BOS participants had both axial and appendicular hypo and hypertonia across the lifespan. Hypotonia was most notable in BRS. On gait, BOS showed difficulty with ambulation without supports, eversion of feet, and lower extremity spasticity (see Figure 2). Only one patient with BOS had normal gait on physical examination. BRS participants had a wide based, clumsy gait. Normal gait exam was seen in 6/7 participants – although three additional participants with BRS had dysdiadochokinesia even in the setting of normal gait.

### 3.5. Quantitative gait analysis

BOS showed distinctive differences in all quantitative gait variables compared to individuals with BRS. BOS had (a) slower pace characterized by smaller normalized velocity, cadence, and step length, and (b) less postural control characterized by a wider stride width (Table 5).

**Table 5:** Spatiotemporal gait variables.

Spatiotemporal variable	BOS (ASXL1)		BRS (ASXL3)	
	*n* = 5		*n* = 6	
	*M*	SD	*M*	SD
Pace				
Cadence (steps/min)	110.33	21.25	125.25	32.68
Normalized velocity (cm/s)	0.50	0.24	0.64	0.17
Normalized step length (cm)	0.27	0.08	0.31	0.03
Postural control				
Normalized stride width (cm)	0.17	0.05	0.09	0.06
Variability				
Step length (cm) %CV	22.43	12.68	19.12	4.57
Step time (sec) %CV	18.89	9.63	28.61	17.05
Stride width (cm) %CV	22.97	1.08	89.41	1.07
Gait variability index (GVI)	153.21	12.27	154.70	5.65

Table of spatiotemporal gait variables for BOS (ASXL1) and BRS (ASXL3).

## 4. Discussion

This study conducted direct qualitative and quantitative phenotyping in 15 individuals with Bohring-Opitz Syndrome (BOS- ASXL1) and Bainbridge-Ropers Syndrome (BRS- ASXL3). The data from this study contributes detailed clinical information to the field and previously reported case series and registry data (Schirwani et al., 2021; Russell et al., 2023).

We found that motor impairments are prevalent and pervasive across the two studied ASXL disorders, both with and without ASD. Unique neurodevelopmental and motor findings in our data include a mixed presentation of hypo- and hypertonia in BOS across a lifespan, slower gait, and poorer postural control compared to BRS. The presence of mixed hypo- and hypertonia calls for a narrowed differential diagnosis, as it illustrates both a peripheral and central process of nerve involvement. Mixed motor presentations can be often misdiagnosed as cerebral palsy within young infants and toddlers (Hakami et al., 2019; Pearson et al., 2019); for this reason, clear delineation of physical exam findings and disease-specific patterns can greatly impact appropriate diagnosis and treatment plans. BRS exhibited hypotonia and greater variability in motor skills. Detailed neurologic examination can aid clinicians in appropriate clinical screening and determination of interventions (e.g., support for hypotonia).

Thus-far, outside of detailed physical examinations, commonly used tools to quantify motor impairments in ASD are the MABC and DCDQ (Liu and Breslin, 2013; Bhat, 2020; Miller et al., 2021; Liu et al., 2023). We found difficulty in using the total scores for the MABC in the BRS population due to the age of participants and overall motor ability level. Two individuals were too young for scoring utilizing the validated MABC charts, and all other patients received a score of “red” on MABC. One individual was older than the validated age range, but the score obtained clearly fell into the red category and was graded as such. All patients received a score indicating developmental coordination disorder on the DCDQ, although 5 individuals similarly fell outside of the age range (4 were too young, and 1 was too old). There were major limitations in the use of the MABC and DCDQ in this population due to the high rates of Intellectual Disability and some individuals having more profound motor difficulties. For example, the results of the DCDQ offer very little information on the variability of motor differences between the groups. Our findings are in line with prior literature for other conditions with intellectual disability and motor delays (Allen et al., 2017; Wilson et al., 2018a,b). However, these tools did provide important insight into the parental perspective and the effect of motor difficulties on their lives; parents highly viewed the motor difficulties as negatively impacting each child’s engagement, self-esteem, and social interactions, with a possible confounding factor of ASD on these effects within the population of individuals with BRS. Twenty-five percent of participants with BOS and 29 % of participants with BRS had a presumed ASD diagnosis. Within the group with BRS, those with presumed diagnosis of ASD were the only individuals whose parents reported “a great deal” of motor difficulties affecting recreational activities (Table 3). On the DCDQ, lower average scores (or more suspicion for developmental coordination disorder) across each subdivision of the assessment was consistently seen for individuals with both BRS and a diagnosis of autism, though this pattern was not seen across subgroups within BOS.

This data emphasizes the importance of motor skills interventions in order to indirectly support other areas of development. As noted from the valuable feedback we collected from the MABC parental forms, parent report can help elucidate the indirect social and cognitive effects of motor development particularly in individuals with genetic neurodevelopmental disorders.

There are limitations in this study which should be noted. The majority of individuals within our study identified as white. The study sample was limited to those individuals and families who were able to travel for the ASXL conference at UCLA, and thus may present both a potential sample bias of individuals whose motor abilities allow them to travel, as well as a bias towards families with the resources to allow them to travel. Some families reported ASD symptomatology but were not given a formal diagnosis. It is possible that ASD is underdiagnosed in this sample given that the symptoms of the condition may be overshadowed by developmental delays and the receipt of a genetic diagnosis (DiStefano et al., 2020). Our results regarding the reported ASD diagnosis and the relationship to motor difficulties should be interpreted with caution. Our findings on the association of motor impairments and presumed ASD diagnoses would be strengthened if we had evidence of a formal diagnostic report confirming ASD diagnosis and/or had conducted standardized assessments measuring ASD symptoms. For these reasons, we present our data as presumed diagnoses of ASD and a preliminary examination of the association of motor impairments and the context of an ASD diagnosis. Our data reflect a cross sectional time point, and it is possible the rates of ASD and other neurodevelopmental disorders may be higher as these participants receive continued evaluation. In planning for future interventions, one of the key components for successful clinical trials will be the discovery of meaningful endpoints in these disorders. As noted above, the floor effects in MABC and DCDQ may limit the monitoring of progress; however, with more quantitative, objective, and finite tools such as the gait analysis in our study, we can aim to have clearer analysis of motor patterns over time. As noted in our study, unique differences can be seen between groups when examining features of each step and stride; and a gross tool to summarize all features such as a GVI can be useful to get more detailed information. Through this combination of individual and summation components of gait analysis, we can work towards a clearer understanding of each disorder’s unique motor presentation, and have objective markers to follow upon clinical intervention initiation. A combination of objective analytics, as well as parental perspective to assist in defining meaningful change in the individual’s life, can help us work towards more effective clinical progress in genetic neurodevelopmental conditions.

## Data availability statement

The data presented in this study are available on request from the corresponding author. The data are not publicly available due to ethical reasons.

## Ethics statement

The studies involving humans were approved by the UCLA Institutional Review Board (IRB#18–000280). The studies were conducted in accordance with the local legislation and institutional requirements. Written informed consent for participation in this study was provided by the participants’ legal guardians/next of kin. Written informed consent was obtained from the individual(s) for the publication of any potentially identifiable images or data included in this article.

## Author contributions

RW conceptualized the design of the study. JA organized the database. MA wrote the first draft of the manuscript and performed the phenotyping categorization, as well as creation of the majority of tables and figures, with the guidance of RW and the assistance of JA. RW and BR edited the manuscript and wrote the sections of the manuscript. All authors contributed to manuscript revision, read, and approved the submitted version.
